# Cotton fiber as a model for understanding shifts in cell development under domestication

**DOI:** 10.3389/fpls.2023.1146802

**Published:** 2023-03-02

**Authors:** Josef J. Jareczek, Corrinne E. Grover, Jonathan F. Wendel

**Affiliations:** ^1^ Department of Ecology, Evolution, and Organismal Biology, Iowa State University, Ames, IA, United States; ^2^ Biology Department, Bellarmine University, Louisville, KY, United States

**Keywords:** cotton fiber, fiber development, fiber evolution, fiber initiation, fiber elongation

## Abstract

Cotton fiber provides the predominant plant textile in the world, and it is also a model for plant cell wall biosynthesis. The development of the single-celled cotton fiber takes place across several overlapping but discrete stages, including fiber initiation, elongation, the transition from elongation to secondary cell wall formation, cell wall thickening, and maturation and cell death. During each stage, the developing fiber undergoes a complex restructuring of genome-wide gene expression change and physiological/biosynthetic processes, which ultimately generate a strikingly elongated and nearly pure cellulose product that forms the basis of the global cotton industry. Here, we provide an overview of this developmental process focusing both on its temporal as well as evolutionary dimensions. We suggest potential avenues for further improvement of cotton as a crop plant.

## 1 Introduction

Cotton is the most widely used plant textile in the world, derived from single-celled epidermal seed trichomes produced by four domesticated species in the genus *Gossypium*. In addition to these four species, the genus is both species-rich and highly variable morphologically, with over 50 species spread across the tropics and subtropics of the world (Wendel and Grover, 2015; Hu et al., 2021). All of these species produce varying degrees of fiber (long, strong seed hairs used in seed dispersal and textile production) and/or fuzz (shorter hairs unsuitable for textiles), with a few notable exceptions, such as the Australian species that produces fat bodies on the seed (Applequist et al., 2001; Wendel and Grover, 2015; Hu et al., 2021). The broad evolutionary relationships among these species are well documented, with *Gossypium* divided into 8 monophyletic diploid genome groups (designated A-G and K) and a single monophyletic allotetraploid group (AD) (Wendel and Grover, 2015; Hu et al., 2021). This latter group includes *G. hirsutum*, or Upland cotton, which is the most widely-grown textile crop in the world (90% of commercial cotton production; Fang, 2018).

**Figure 1:**
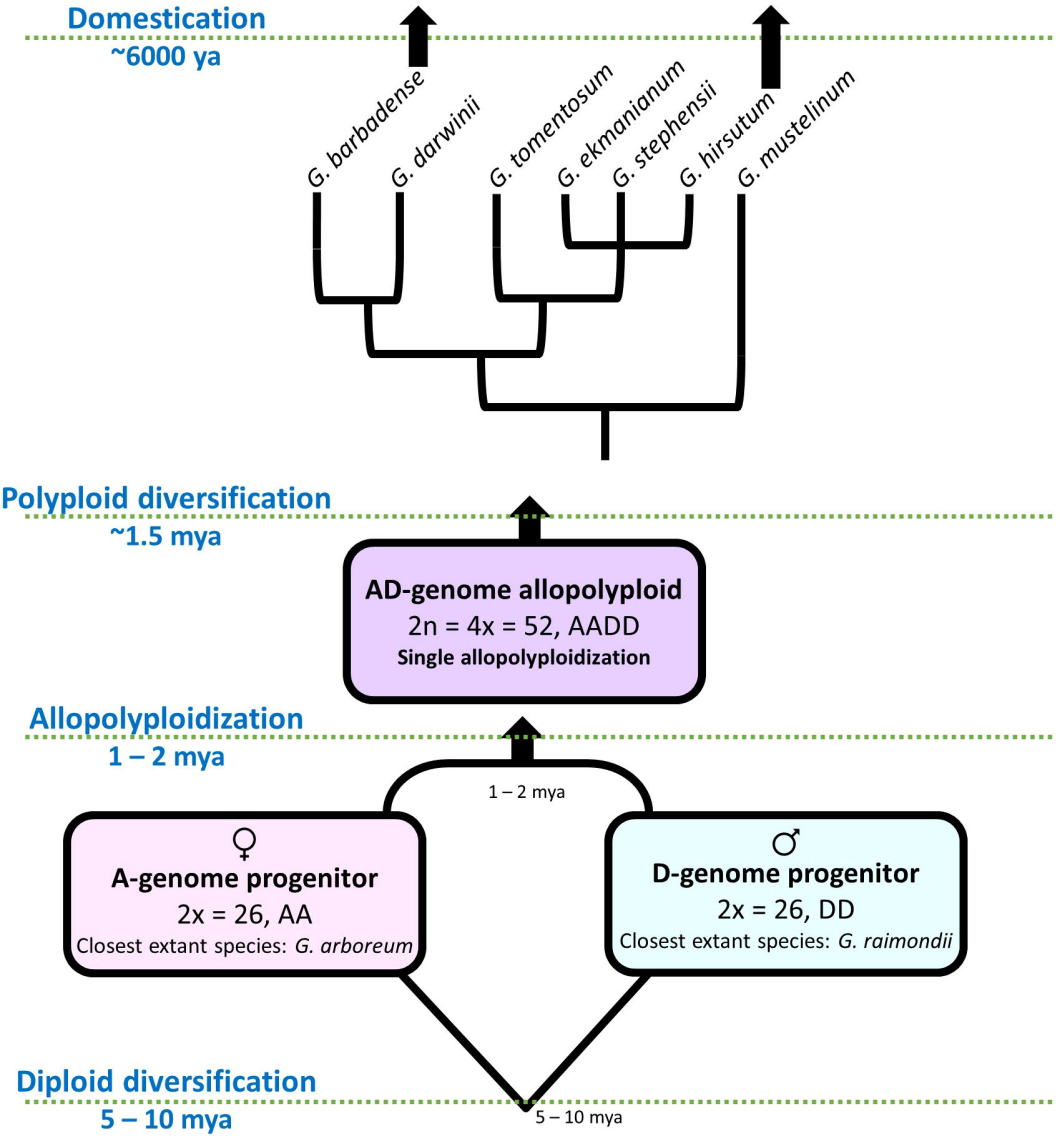
Evolutionary model for allopolyploid *Gossypium*. Approximately 5-10 mya, the cotton underwent diversification resulting in separate evolutionary trajectories for the now-extinct A-(maternal) and D-(paternal) progenitors of the allopolyploid clade. Subsequently, (~ 1 mya) these lineages hybridized and underwent genome doubling, resulting in the allotetraploid AD clade. This clade diversified into seven polyploid species, two of which underwent domestication and improvement, *i.e., G. hirsutum* and *G. barbadense*.

Beyond the value of cotton as a crop species, it has also been developed into a model system for studying polyploidy and domestication (**Figure 1**). Although at present *G. hirsutum* and a second allotetraploid species (i.e., *G. barbadense*, or Pima Cotton) together comprise about 93% of all cotton fiber produced worldwide, cotton has been independently domesticated four times from four different wild species. The genus originated approximately 5-10 million years ago, with the basal-most radiation resulting in the separation of the two lineages that would later comprise the allotetraploid (*i.e.*, the A and D genomes). Approximately 1-2 million years ago (mya), now extinct members of the A and D lineages were reunited in a common nucleus to form the allotetraploid (AD) lineage, which subsequently diverged into the seven recognized polyploid species of *Gossypium* (AD1-AD7) (Wendel et al., 2010; Grover et al., 2015; Wendel and Grover, 2015; Gallagher et al., 2017; Hu et al., 2021). Along with the two aforementioned domesticated allotetraploids resulting from this single polyploidization event (i.e., *G. hirsutum*, AD1 and *G. barbadense*, AD2), the two extant diploid species from the A-genome (*G. herbaceum*, A1 and *G. arboreum*, A2) were also independently domesticated by humans approximately 5-8 thousand years ago, all of which are still grown on various scales (Wendel et al., 2010). Notably, while the domesticated allotetraploids partly derive from a member of the (later) domesticated A-genome, factors from the short-fibered D-genome also contribute, more or less equally, to the agriculturally important fiber phenotype (Bao et al., 2019; Guo et al., 2021; Hu et al., 2021; Zhu et al., 2021; Lu et al., 2022). This observation demonstrates that an ancient allopolyploidy event that occurred prior to human evolution enabled humans to develop and select cotton fiber with vastly improved properties to those found in nature. It is this combination of a naturally replicated domestication experiment across four species and two ploidy states that makes *Gossypium* a powerful system to study both of these phenomena, as well as the underlying genetics, genomics, and genotype-to-phenotype transitions.

Although cotton has multiple economic uses in addition to textiles, the primary reason behind its domestication and continued economic importance is its ability to produce fiber, the long, strong, single-celled trichomes or hairs that arise from the seed epidermal layer (Lee et al., 2007; Hu et al., 2016a). Although all wild species of cotton produce fiber, domestication has in all cases generally led to longer, stronger, and whiter hairs with varying degrees of fineness (here, defined as unit weight per unit length, as per Ramey (1982) and Kelly et al. (2015). It has also led to increased susceptibility to various biotic and abiotic stresses. *G. barbadense* fiber is used to produce a well-known luxury textile (e.g., Pima, Sea Island, and Egyptian cotton) for the fineness, length, and strength of the product it produces; however, yield is lower and the plants are not as well-adapted to diverse environments compared to the coarser and weaker fiber produced by the dominant crop species, *G. hirsutum* (Constable et al., 2015). In addition, *G. hirsutum* is more easily grown and generally is more resistant to pests and disease, requiring fewer resources (i.e., pesticides and fertilizers) to grow than *G. barbadense* (Constable et al., 2015). Similarly, whereas the two domesticated diploids produce fiber that is inferior to that of either allotetraploid, these species are still locally grown in regions of South Asia and the Middle East, due to their adaptation to local and regional growing conditions and pests (Constable et al., 2015).

**Figure 2:**
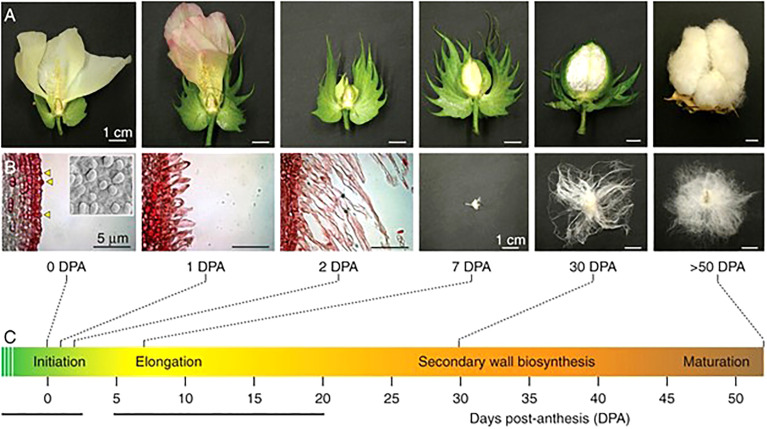
A depiction of fiber development showing both macro- **(A)** and microscopic **(B)** views of G. hirsutum fiber at several important timepoints, described in C. At 0 DPA, fiber initials are present on the surface of the ovule. From 1-2 DPA, fiber tips are refined and elongation begins. By 7 DPA, all fibers are undergoing elongation. Elongation continues and subsequently begins to taper in the transition stage (~14 DPA, depending on species and/or accession) as secondary wall synthesis begins. By 30 DPA, elongation has ceased and the fiber is actively engaged in secondary cell wall deposition. The fruit finally matures at around 50 DPA, at which point the mature fibers have died and the boll dehisced. Scale bars for the scanning electron images is 5mm; all other scale bars are 1cm. The original picture is from Lee et al. (2007), reprinted with permission.

Because it is not only the presence of fiber, but also the properties of fiber that influence its economic value, the mechanisms underlying physical transformation from fiber initial to mature fiber are of considerable interest. The development of the cotton fiber can be divided into overlapping but biologically determined stages (**Figure 2**), beginning with initialization on the ovule surface prior to anthesis (Applequist et al., 2001; Butterworth et al., 2009; Kim, 2015; Kim, 2018). After fertilization, the fiber initial undergoes tip refinement and transitions to the elongation phase, which can continue until as late as 25 days post anthesis (DPA) in domesticated accessions (Applequist et al., 2001; Haigler et al, 2012; Kim, 2018; Graham and Haigler, 2021). During elongation, the fiber remains expandable, growing to a length of up to 6cm (Kim and Triplett, 2001; Butterworth et al., 2009). At approximately 15DPA, the fiber enters the transition stage as elongation slows and stops while the secondary cell wall deposition stage begins (Haigler et al., 2012; Kim, 2015; Kim, 2018). The transition phase lasts from approximately 15-20 DPA, after which the fiber is fully committed to secondary wall synthesis, which thickens the secondary cell wall until roughly 40 DPA, or maturity (Haigler et al., 2009; Haigler et al., 2012; Stiff and Haigler, 2012; Kim, 2018). During this final active stage of development,

**Figure 1** A model of *Gossypium* evolution. Approximately 1 mya, divergent A and D lineages hybridized and underwent genome doubling, resulting in the allotetraploid AD clade. This clade diversified into seven polyploid species, two of which underwent domestication and improvement.

Cellulose fibers are laid down in a helical pattern around the fiber cell, forming a thick, strong cell wall that is critical to the quality of the fiber (Haigler et al., 2009; Tuttle et al., 2015). Once mature, the fiber undergoes cell death and the cytoplasm is degraded, leaving behind a hollow cellulose tube that takes on a distinctive kidney bean shape in fiber cross-sections. The mature fibers dry out and naturally twist, which makes them easily spun into threads and fabric, and the fiber holds dye well (Constable et al., 2015; Tuttle et al., 2015).

Underlying this developmental process are coordinated changes in gene expression and all of the “omics” that propagate through complex networks of hormone signaling, physiology, and biosynthesis. These patterns ultimately determine the final properties of the mature fiber, including the agronomically important traits of strength, length, and fineness. Here we present an overview and update what is known about cotton fiber development at each of these stages (i.e., initiation, elongation, transition, cell wall thickening, and fiber maturation) and how differences in these stages result in fiber with different properties at maturity. We consider potential avenues of future research in cotton, and discuss how a deeper understanding of fiber development may lead to improvement in cotton as a textile. We also apply an evolutionary lens, highlighting what might be learned from carefully studying the natural and genomic context of the wild species in the genus.

**Figure 2** A depiction of fiber development showing both macro- and microscopic views of *G. hirsutum* fiber at several important timepoints. At 0DPA, fiber initials are present on the surface of the ovule. From 1-2 DPA, tip refinement takes place and fiber elongation begins. At 7 DPA, fibers are elongating. Around 14D DPA, the fiber enters the transition stage. By 30 DPA, elongation has ceased and the secondary cell wall has been laid down. By 50 DPA, the mature fibers have died, and the boll dehisced. Scale bars for scanning electron images is 5μm. All other scale bars are 1cm. Original picture Lee et al., 2007 with permission.

## 2 Phase 1, 0-4 DPA: Initiation and tip refinement

Cotton fiber is initialized on the surface of the ovule before anthesis. Initials begin as spherical protrusions of epidermal cells on the chalazal end of the cotton ovule, with fibers initializing towards the micropylar end over the first day post anthesis (Butterworth et al., 2009). Once fiber initials are established, they undergo tip refinement (see below) and elongation, with these processes co-occurring for all initials in domesticated *G. hirsutum* (Lee et al., 2007; Butterworth et al., 2009; Kim, 2015). Conversely, these processes differ somewhat in wild cotton, which have lower percentages of fiber initials on the surface of the ovule; tapering occurs later in *G. herbaceum*, and has a wider variety of fiber lengths and widths during this time (Butterworth et al., 2009). During initiation in domesticated species, the fiber also produces the cotton fiber middle lamella (CFML), an adhesive layer that joins together elongating fibers into bundles that remain in this state until the transition stage, where the CFML is broken down and fibers are released to develop individually again (Singh et al., 2009; Haigler et al., 2012). The CFML has not been well-studied in wild *Gossypium*. It is thought that the CFML aids in fiber organization during elongation, keeping the fibers in distinct bundles and preventing the fibers from elongating in random directions (Singh et al., 2009). This allows the fibers to grow longer in the confined space of the locule.

The process of tip refinement is particularly important for fiber quality, as tip refinement is strongly correlated with fiber diameter, which is in turn strongly correlated with the properties of mature fiber. The ratio of the fiber diameter to the fiber lumen is directly related to fiber strength, dyeability, and spinnability (Kelly et al., 2015). Tip refinement is accomplished by the concerted synthesis of the cell wall and cytoskeleton, with fiber tips being tapered during the first day post anthesis (Graham and Haigler, 2021). Graham and Haigler (2021) demonstrated that microtubule arrays present near the apex of the fiber were necessary for proper fiber development, and when affected by microtubule antagonists during tip refinement, fiber tips and shape were partially disrupted, resulting in fibers with a larger diameter and irregular shapes (Graham and Haigler, 2021).

There are three potential fiber types based on tip morphology (narrow, tapered, hemisphere/blunt) that confer slightly different characteristics, most notably fiber diameter, which tends to be narrower in tapered versus blunt tips (Graham and Haigler, 2021). *G. hirsutum* possess two of the fiber types, which have either blunt/hemisphere tips or tapered tips (Stiff and Haigler, 2016), whereas *G. barbadense* has the third type (narrow). While also tapered, experiments suggest that the narrow tips in *G. barbadense* are a distinct cell type from those in *G. hirsutum*, conferring a smaller fiber diameter (Stiff and Haigler, 2016; Graham and Haigler, 2021). Notably, the two distinct tip morphologies in *G. hirsutum* produce heterogeneous fibers on single seeds and hence bolls that have slightly different characteristics; specifically, the hemisphere tipped fibers have a twofold larger apical diameter than the tapered tips, which may ultimately influence fiber length and strength (Stiff and Haigler, 2016; Graham and Haigler, 2021). Additionally, the presence of two populations of fiber tip types may contribute to lower fineness in *G. hirsutum*, as many of the fibers have a higher diameter. The single narrow tip morphology in *G. barbadense* leads to homogeneous fiber with a more uniform and smaller diameter that may contribute to its higher quality (Stiff and Haigler, 2016). While tip refinement in the diploid domesticated species is less well-studied, a few accessions analyzed thus far have hemisphere-tipped fiber initials, leading to higher-diameter fiber (Butterworth et al., 2009).

Despite the distinct diameter sizes observed for these three fiber types at 4DPA, they achieve similar rates of elongation during the first four days post-anthesis (Graham and Haigler, 2021). During this time period, however, tip shape is being refined differently. While the budding fibers in domesticated *G. hirsutum* have similar tip shapes at 1 DPA, they subsequently establish two fiber tip subpopulations over the next day. In *G. barbadense*, tips also begin tapering at 1 DPA and continue to do so uniformly through 2 DPA. Interestingly, while tip tapering occurs at different times of the day in *G. hirsutum* and *G. barbadense* (morning and evening, respectively), final taper morphology is established in both by the second morning post-anthesis (Graham and Haigler, 2021).

Taken together, these observations regarding fiber initiation and tip refinement raise questions about cotton fiber tip evolution, across both ploidy and domestication states: why does *G. hirsutum* have two tip morphologies? What is the basal state? More research into the effects of domestication, and into tip morphology of the diploid fiber-producing cottons, may provide insight into how this interesting difference evolved.

The initiation phase is regulated by several phytohormones, including brassinosteroids, abscisic acid, and jasmonic acid, and by auxin, which is transported into the fiber cells from the ovule (Ahmed et al., 2018; Xiao et al., 2019). Auxin plays a key role in fiber initiation, with excess auxin application (exogenous or endogenous) increasing the number of fiber initials on the surface of the ovule, and reduction of auxin transport into the fibers inhibiting fiber initiation (Xiao et al., 2019). Brassinosteroids have been shown to regulate fiber initiation as well; that is, when BR biosynthetic or signaling pathways are disrupted, few fibers initialize on the ovule surface (Ahmed et al., 2018; Xiao et al., 2019). Abscisic acid has been shown to inhibit fiber initiation, with increased ABA concentration directly correlating with fewer fiber initials (Xiao et al., 2019). Jasmonic acid has been reported to control fiber initiation as well, by degrading highly-expressed jasmonic acid negative regulators and switching on pathways related to trichome development (Wang et al., 2015; Xiao et al., 2019). GWAS studies reveal loci that impact initiation as well, and genes such as *GhCIP1* (an F-box interacting gene shown to play a role in flowering time (Ma et al., 2018)), and *GhUCE* (a ubiquitin-conjugating enzyme (Ma et al., 2018)) that potentially regulate initiation and yield (Yang et al., 2020).

Studies have been conducted exploring genes important during the initiation stage. WRKY16, for example, is a transcription factor (TF) shown to promote fiber initiation by promoting expression of other TFs, including those with broad regulatory functions such as MYB109 and MYB25 (Wang et al., 2021). When these transcription factors were silenced, cotton ovules initiated fewer fibers, and fibers were significantly shorter (Wang et al., 2021). Extensive research has been done to determine other TFs and genes important for fiber initiation, including functional studies (Wang et al., 2020). These functional studies are varied; some examples include those that impact transcription factors, such as silencing (via RNAi) and overexpression of MYB25, showing reduced fiber initiation and elongation and increases in initiation, respectively (Machado et al., 2009) and expression of a *Gossypium* bHLH TF in *Arabidopsis* leading to increased trichome initiation on leaves and stems (Shangguan et al., 2016). Altering hormone regulatory genes also impacts this stage of development; overexpressing auxin biosynthesis *via iaaM* increased levels of initiation and of lint fiber specifically (Zhang et al., 2011), and silencing PIN1a to suppress auxin transport resulting in lower fiber elongation (Zhang et al., 2011). These are not the only two areas that impact initiation and tip refinement; RNAi of *GhHDA5*, a histone deacetylase, resulted in reduced initiation, alterations in reactive oxygen species (ROS) management, and increased autophagy in fiber (Kumar et al., 2018); and suppression of sucrose synthase leading to lower initiation and elongation in the fiber (Ruan et al., 2003). In short, fiber initiation is a complex process involving many aspects of cell development, including hormonal regulation, ROS signaling, and gene suites regulated by high-level transcription factors.

The initiation stage of cotton development is undergoing a recent surge in study, and contains important implications for fiber improvement. Uncovering the mechanisms by which fiber diameter is determined during this early stage, or perhaps discovering methods by which hemisphere tipped fibers in *G. hirsutum* could be converted to tapered tipped fibers could result in longer, stronger fibers with increased fineness in elite lines. Likewise, changing the amount of fiber that is initialized on the surface of the ovules through hormone manipulation, particularly in low-producing cultivars, may lead to higher yields, although this would need to be balanced with physical constraints within the locule. Comparison between wild and domesticated accessions, and among species, may provide the insight into the fiber initiation program necessary to make these improvements, particularly in the understudied diploid species. However, careful study will be necessary to maximize improvements gained from these possibilities while also elucidating potential trade-offs in fiber quality or broader impacts on the plant at large, such as resource needs (carbohydrates in particular). Care must also be taken to ensure that higher yield does not come at the cost of lower quality; increasing the number of initials on an ovule may result in a larger harvest, but that is no guarantee that the fibers produced will be of high quality (Bradow et al., 1997; Davidonis et al., 2004).

## 3 Phase 2, 3-25 DPA: Elongation

As the fiber is finalizing tip refinement, it continues to undergo anisotropic growth and elongate (i.e., the elongation phase), eventually achieving its mature length by roughly 14 DPA in wild species and 18-25 DPA in domesticated species (Applequist et al., 2001). Of note, this extended elongation in domesticated species appears to be the result of human selection (Applequist et al., 2001). During this time, the fiber undergoes primary cell wall synthesis and linear growth until it reaches its full length, which can be as long as 6 cm in elite domesticated lines (Kim and Triplett, 2001; Butterworth et al., 2009). The primary cell wall of *G. hirsutum* and *G. barbadense* fiber is fairly typical, consisting mainly of cellulose, pectins, and xyloglucan (Haigler et al., 2012; Avci et al., 2013). Domesticated diploid species are less well-studied, but research indicates that they have similar primary cell walls as well (Huwyler et al., 1979; Tokumoto et al., 2002). The final composition of the primary cell wall is approximately 22% cellulose, surrounded by xyloglucan and pectin – a drastic difference from the secondary cell wall, discussed below (Haigler et al., 2012).

Cotton fibers are believed to expand using a linear growth mode, which combines both diffuse and tip growth to produce the typical anisotropic growth observed in fiber (Kim, 2018). During this period, the fiber exhibits high turgor pressure and has high expression levels of expansins, i.e., proteins responsible for cell wall loosening (Haigler et al., 2012; Kim, 2018; Yaqoob et al., 2020). The direction of expansion is guided by regulating the flexibility of the primary cell wall, which is done through cellulose deposition that follows microtubule arrangements in the fibers (Haigler et al., 2012; Kim, 2015; Graham and Haigler, 2021). The actin cytoskeleton, along with a host of actin-interacting genes such as profilin, villin, actin depolymerizing factor, the actin-related protein 2/3 complex (an actin nucleator), and others, also play an active role in fiber elongation by directing cell wall component deposition (Staiger et al., 1997; McCurdy et al., 2001; Sheahan et al., 2004; Wang et al., 2005; Augustine et al., 2008; Suarez et al., 2015). Interestingly, plasmodesmata are known to also play a role in elongation by closing and opening during development to assist in solute transport or to maintain turgor pressure in the developing fiber (Ruan et al., 2001; Ruan et al., 2004). Finally, reactive oxygen species (ROS) management also plays an important role in fiber elongation (Li et al., 2007; Hovav et al., 2008; Chaudhary et al., 2009; Tang et al., 2014; Tuttle et al., 2015; Xu et al., 2019). It has been shown that calcium-based ROS management has a direct impact on elongation; in one example, overexpressing certain calcium sensors promotes fiber elongation, as does the application of exogenous hydrogen peroxide, a common ROS and one known to play a role in cell elongation (Hovav et al., 2008; Chaudhary et al., 2009; Tang et al., 2014). More recently, an in-depth analysis of ROS networks in *G. hirsutum*, *G. arboreum*, and *G. raimondii* showed that several ROS management gene families participate in the elongation, transition, and cell wall thickening stages, ensuring that ROS levels are maintained at levels appropriate for each stage (Hovav et al., 2008; Chaudhary et al., 2009; Xu et al., 2019).

While elongation continues past the initiation of secondary cell wall deposition, it slows considerably during the transition stage (16-20 DPA). Notably, elongation can continue in *G. barbadense* until as late as 25 DPA, which is three to five days longer than in *G. hirsutum*, where elongation typically ends around 20-22 DPA (Applequist et al., 2001; Chen et al., 2012). In wild species, the elongation period is shorter, ending at roughly 14 DPA (Applequist et al., 2001). This extra time spent in elongation is thought to be part of the reason that *G. barbadense* fibers are higher quality; they are longer than their counterparts in *G. hirsutum* (Chen et al., 2012). The combination of a narrower diameter due to tip refinement and a longer fiber due to extended elongation leads to finer fiber.

The cell wall is deposited in a typical fashion, with the actin cytoskeleton playing an important role in delivering materials to the site of elongation and providing structure to the elongating fibers (Seagull, 1990; Li et al., 2005; Lv et al., 2017; Fang et al., 2018; Pandey and Chaudhary, 2019). The microtubule cytoskeleton serves to guide the deposition of cellulose in the primary cell wall as well as guiding fiber diameter as discussed above (Paredez et al., 2006; Li et al., 2012; Graham and Haigler, 2021). Between the tight regulation of cell wall development during the elongation phase and the high turgor pressure of the cells, cotton achieves the strong anisotropic growth pattern that results in a long, single-celled fiber.

Many of the major plant hormones play a role in elongation, whether by promoting or inhibiting the growth of the fibers. As with initiation, auxin plays a pivotal role during fiber elongation, in part contributing to the loosening of the cell wall during elongation (Chen et al., 2012; Ahmed et al., 2018). Application of exogenous auxin can increase fiber length, whereas interfering with auxin signaling leads to shorter fibers (Xiao et al., 2019). Another phytohormone, gibberellic acid, has been repeatedly shown to improve not only fiber length (by regulating cell wall development during the elongation stage), but also fiber strength and fineness (Aleman et al., 2008; Bai et al., 2014; Zhang et al., 2017b; Ahmed et al., 2018; Xiao et al., 2019). Brassinosteroids are also known to be required for fiber elongation; when BR signaling or biosynthesis is disrupted, fiber length is reduced and can be rescued *via* BR application (Ahmed et al., 2018; Xiao et al., 2019). Conversely, high concentrations of abscisic acid have been shown to inhibit fiber elongation (Ahmed et al., 2018), although the mechanism by which this occurs is unclear.

Ethylene, however, arguably is the most important hormone for fiber elongation. Many ethylene biosynthetic genes are upregulated during elongation, as are ethylene signaling pathways (Ahmed et al., 2018; Xiao et al., 2019). Application of ethylene results in longer fibers, and increases the expression of genes known to be involved in fiber development and growth, such as sucrose synthase, cellulose synthase, and expansins. (Ahmed et al., 2018; Xiao et al., 2019). Ethylene also interacts with the BR pathway and plays a crucial role in ROS and Ca^2+^ management, both of which are key processes during elongation (Ahmed et al., 2018; Xiao et al., 2019). ROS regulation in particular is known to impact cell wall extensibility, which is carefully regulated to ensure the fibers elongate anisotropically (Tang et al., 2014).

The elongation stage is a popular target for functional studies and molecular work in *Gossypium*, as fiber length is the most visible trait for fiber. Examples of notable genes and studies examining this stage include: suppression of *GhMYB109*, a MYB TF, revealed that it is required for fiber elongation (Pu et al., 2008); MYB25, discussed above, also results in shorter fiber when suppressed (Machado et al., 2009); overexpression of *GhHOX3*, a homeodomain-leucine zipper TF, increased fiber length, and RNAi resulted in shorter fibers (Shan et al., 2014); suppressing a number of hormone signaling or biosynthesis-related genes (including jasmonic acid, gibberellin, brassinosteroid, and auxin) produced shorter fibers (Luo et al., 2007; Yang et al., 2014; Hu et al., 2016b; Zhang et al., 2017a); and several studies have been done over the years describing the importance of various cytoskeletal and cell wall genes for fiber elongation, as reviewed in (Huang et al., 2021). Additionally, a notable mutation in an actin gene is responsible for the well-known *Ligon-lintless 1* mutant, which has a short-fiber phenotype, demonstrating the importance of actin organization in elongation (Li et al., 2005; Kim, 2015; Takatsuka et al., 2018). Several QTL in *G. hirsutum* that impact fiber elongation, as discussed in (Dong et al., 2018; Liu et al., 2018; Liu et al., 2019; Naoumkina et al., 2019; Xu et al., 2020), and others.

Elongation is a clear focal point for fiber improvement, as elite cotton lines must have long fiber near the top of the known range. As with fiber initiation and tip refinement, alterations here could produce unwanted side effects in fiber strength (a long fiber without the proper reinforcement of the secondary cell wall is weak), but extending the elongation stage of elite lines may be a potential avenue for improvement. Finding ways to alter the primary cell wall composition for more desirable extensibility could lead to higher fiber quality as well (Kelly et al., 2019; Mathangadeera et al., 2020). Fine-tuning the developmental balance of key regulatory proteins and those governing the levels of growth hormones is another potential method of improvement. Further study into the mechanisms of how hormones impact elongation and how they interact with one another during this stage may provide insight into how to manipulate fiber length.

## 4 Phase 3, 16-20 DPA: Transition

The transition phase refers to the period during which the fiber switches from elongation to secondary wall synthesis. It is a time where the fiber is coordinating significant alterations to its transcriptome and cellular processes to begin the delicate interplay between cell elongation and cell wall thickening. As the fiber reaches ~16DPA, elongation and primary cell wall deposition begin to slow and stop, ceasing in all species by 25 DPA (Chen et al., 2012; Tuttle et al., 2015). While elongation slows, microtubules within the fiber shift to a helical angle and the fiber lays down a transitional winding cell wall layer (Meinert and Delmer, 1977; Seagull, 1993; Hsieh et al., 1995). This layer is thin and flexible and has a composition similar to that of the primary cell wall, with a small increase in cellulose content (Tuttle et al., 2015). Notably, the angle of cellulose deposition changes in this layer from a transverse orientation to a shallow helix (Graham and Haigler, 2021); this change in angle is important, as it likely leads to increased fiber strength (Haigler et al., 2009; Tuttle et al., 2015; Nixon et al., 2016; Zhang et al., 2021).

Secondary cell wall deposition begins during the transition stage, thickening the fiber through the next 20-30 days and resulting in a cell wall consisting of ~98% pure cellulose, a composition that is remarkable among plant cell walls (Kim, 2018). The transition phase is characterized by extensive changes in gene expression and phytohormone regulation, most notably auxin and gibberellic acid, which play an important role in the transition, along with changes in the regulation of various reactive oxygen species in the fiber (potentially altering fiber extensibility, as discussed above) (Tuttle et al., 2015; Xiao et al., 2019; Zhang et al., 2020).

The transition phase is characterized by massive regulatory changes that herald the shift from fiber elongation to the tightly regulated program required for cell wall thickening. Naturally, these sweeping transcriptional changes often are related to expression changes in various stage-specific transcription factors. Broad-scale surveys have identified several NAC-domain factors as important, such as SND1 and TALE family genes, which activate secondary cell wall deposition when upregulated (Zhong et al., 2006; Ma et al., 2019). Altered expression of various MYB-domain transcription factors is also implicated during the transition stage, leading to expression changes across all downstream genes (Zhong et al., 2006; Li et al., 2009). GhMYBL1, for example, is known to be expressed during this stage, and when overexpressed in *Arabidopsis*, causes enhanced secondary cell wall synthesis in xylary elements (Sun et al., 2015). While much of this understanding comes from broad-scale surveys, some research has been done on specific transcription factors as well. For example, GhTCP4 overexpression promotes secondary cell wall thickening, resulting in shorter, thicker fibers (Cao et al., 2020). Hot216, a transcription factor that encodes a KIP-related protein, plays a role in the transition stage as well, regulating a network of nearly 1000 genes related to cell wall synthesis (Li et al., 2020).

As a result of these massive transcriptional changes, the transition phase is also marked by dramatic proteomic and metabolomic shifts. Both *G. hirsutum* and *G. barbadense* exhibit increases in cytoskeletal, carbohydrate metabolism, and redox proteins during this stage (Zhou et al., 2019). Both species also exhibit simultaneous decreases in proteins responsible for the biosynthesis of many common cell wall polysaccharides, to better direct metabolite flux into the cellulose synthesis pathway (Zhou et al., 2019). For example, during the transition stage, the fiber transcriptionally modifies a pathway similar to that of sclerenchyma differentiation in *Arabidopsis*, to repress lignin biosynthesis while upregulating cellulose biosynthesis in the fiber (Tuttle et al., 2015). This is what causes the nearly-pure cellulose phenotype seen in mature fiber secondary cell wall (SCW). The lignin pathway is not entirely repressed, however; small amounts of lignin precursor compounds are synthesized in the fiber, which likely account for the presence of lignin-like phenolics in the mature fiber (Tuttle et al., 2015). It is also during this stage that the CFML breaks down, allowing the fiber bundles to separate into individual fibers.

Because of the significant and important changes that occur during the transition stage, it requires careful study before it can be targeted for improvement. The confluence of the elongation stage and the secondary wall synthesis stage provide an opportunity to fine-tune the relationship between the two, which may in turn impact fiber strength and fineness. Understanding the transition stage is critical for fiber improvement, as it is where these two fiber qualities begin to compete. The end of elongation and the beginning of secondary wall synthesis also present opportunities to deepen understanding of these two processes, which could in turn lead to further improvements. Extending elongation, for example, is one way in which the domesticated cotton phenotype was impacted by human selection (Applequist et al., 2001). The deposition of the winding cell wall layer may provide an avenue for improvement, as it is a contributor to the final strength of the fiber and an important component of the fiber cell wall.

## 5 Phase 4, 20-50 DPA: Secondary cell wall thickening, maturation, and cell death

The final stage in fiber development, generally referred to as secondary wall synthesis, is the stage that determines the width of the fiber cell wall along with further impact on fiber strength. Cellulose synthesis begins to increase around 14DPA, and by ~25DPA, the fiber across all species has entered secondary cell wall thickening. Here, the fiber lays down cellulose in a steep helical pattern, guided by microtubules (Seagull, 1990; Seagull, 1993). In contrast with wild cotton fiber, which contains lignin and other cell wall polysaccharides, domesticated cotton fiber is nearly pure (~98%) cellulose (Haigler et al., 2009; Haigler et al., 2012; Tuttle et al., 2015). This tube of nearly pure cellulose, deposited in a helical pattern, is all that remains after the fiber undergoes cell death, providing strength and spinnability through the fine control of wall thickness (Tuttle et al., 2015).

At the molecular level, cellulose synthases (CESAs) are upregulated during the stage; specifically, a subgroup of the 32 *Gossypium* CESA genes are expressed during SCW synthesis (Kim, 2018). As in most plant cellulose synthase complexes (CSCs), these CESA subunits are arranged into a rosette consisting of six linked trimers, or 18 CESA subunits (Nixon et al., 2016; Kim, 2018). These CSCs lay down cellulose fibrils in a helical arrangement around the cell following the pattern established by the microtubules, but with periodic reversals of the helical orientation (Kim and Triplett, 2001; Betancur et al., 2010; Li et al., 2012; Kim, 2018).

Of the phytohormones, auxin (IAA) and gibberellic acid (GA) have the greatest impact during secondary cell wall deposition. IAA concentration during this stage of fiber development is found to increase, and application of exogenous IAA is found to increase cell wall thickness (Zhang et al., 2011; Xiao et al., 2019). GA also plays a role in secondary cell wall synthesis, as it is a regulator of sucrose synthase genes, which are crucial for cellulose production (Brill et al., 2011; Bai et al., 2014; Xiao et al., 2019).

The onset of secondary cell wall synthesis is accompanied by a transcriptional and proteomic switch, as the fiber ceases elongation and slows or stops the deposition of non-cellulose wall components. Accordingly, the fiber undergoes significant regulatory and gene expression changes. Functional studies have been performed at this level as well, with a focus on cell wall thickening specifically. Some studies report the following: expression of *GhMYB2*, a MYB TF, in *Arabidopsis*, resulting in thicker leaf trichomes (Huang et al., 2013); increasing gibberellin biosynthesis in the fibers through *GA20-oxidase* and *GA2-oxidase 2* resulted in thinner fibers, indicating alterations to fiber thickness (Bai et al., 2014); overexpressing *GhSusA1*, sucrose synthase, resulted in thicker fibers, and suppression of the gene produced thinner fibers (Jiang et al., 2012); in one study, cellulose synthase genes (*acsA* and *acsB*) from *Acetobacter xylinum* were transformed into *Gossypium*, resulting in fiber with thicker, stronger fiber (Li et al., 2004); and FSN1, a NAC TF, was shown to positively regulate secondary cell wall synthesis in *G. hirsutum* (Zhang et al., 2018). QTL studies have also identified QTL that impact fiber thickness and cell wall development, as seen in (Ma et al., 2019; Li et al., 2020; Hafeez et al., 2021) and others.

The process of cell wall thickening continues for several weeks, with cotton fiber typically reaching maturity from 40-50 DPA (Kim, 2018). When the fibers are mature, the fiber cells die, dry out, and the bolls dehisce. As they dry, the fiber is reduced to a hollow tube of cellulose through processes that are not understood (Kim, 2018). The fiber itself collapses into a bean shape in cross section, which contributes to the twisting of the mature fibers. Detailed research on these later DPA during the cell wall thickening or maturation stage is scarce due to the challenges imposed by the thick cell wall. Mechanically breaking the fibers open is difficult, and the strength of chemical degradation required for cell wall lysis also frequently damages the contents of the fiber cell. Consequently, little is known about the processes that follow SCW thickening and/or the mechanisms by which the fiber is converted from a living cell into the characteristic hollow tube of cellulose.

With respect to fiber improvement, optimization of secondary cell wall deposition could contribute to higher quality fiber through modifications of the fiber wall thickness to cell diameter ratio. This does come with the risk of over-thickening the cell wall, leading to coarse, bristle-like fibers that do not spin or dye well, as seen in some wild species. While there may also be potential improvements in the fiber maturation and desiccation aspects, assessment of these is prohibited until further progress is made on understanding fiber maturation, apoptosis, and desiccation. Some data of these processes is available; for example, it is known that desiccation of the boll begins before the boll opens (Lee et al., 2015), but much work remains to be done.

## 6 Insights from evolutionary biology

Cotton fiber derives from a plant with a unique and fascinating evolutionary history spanning millions of years and including several remarkable biogeographic and genomic events. The natural hybridization of an Old World A genome species with a New World D genome species and the subsequent rise of a new clade of allotetraploid AD species provided *Gossypium* with duplicated copies of its genes, which enabled greatly expanded opportunities for mutation and recombination over evolutionary time and novel combinatorial possibilities (Wendel et al., 2010; Wendel and Grover, 2015). This polyploidization was then followed by the independent domestication of four species, two diploid and two polyploid (Wendel et al., 2010; Wendel and Grover, 2015). The strong directional selection of domestication combined with variation in ploidy level provides a powerful lens to examine how these traits have impacted the fiber, along with gleaning insight into how fiber might be further improved.

Polyploidy also plays an important role in fiber development; one of the key features of polyploid organisms is that their genome contains multiple copies of each gene. Some of these copies are lost over time, but some obtain new functions, or split their function between the copies (Soltis and Soltis, 1999; Adams and Wendel, 2005; Otto, 2007; De Smet and Van de Peer, 2012; Wendel, 2015; Fang et al., 2017). Additionally, having more copies of genes means that selection against mutation is relaxed, as deleterious mutations in one copy will have less of an impact as long as the second copy is intact (Adams and Wendel, 2005; De Smet and Van de Peer, 2012). Polyploidy also gives rise to cytonuclear effects – in cotton, the presence of both A- and D-genome genes in an A-genome cytoplasm leads to a variety of *cis*-*trans* interactions not seen in diploids (Bao et al., 2019). These provide opportunities to study the impact of *cis*- and *trans*-regulation on the fiber phenotype, and to consider how it may be leveraged to improve cotton fiber.

In addition to these broad evolutionary concepts, morphological and genetic/transcriptomic studies provide a window into some of the evolutionary processes responsible for cotton fiber as a whole, differences between the fibers of domesticated species, and how domestication produced a very similar phenotype in four independent species. Transcriptomic studies reveal that cotton fibers do not utilize all pathways typically associated with trichomes, but instead include some that are more similar to xylem elements or other sclerenchyma cells in in *Arabidopsis* (Haigler et al., 2009; Betancur et al., 2010; Sun et al., 2015; Tuttle et al., 2015). The appearance of three distinct tip morphologies among *G. hirsutum* and *G. barbadense* fibers point toward unique genomic bases and developmental programmes of domestication between the two species, despite having generally similar fiber phenotypes (Stiff and Haigler, 2016; Graham and Haigler, 2021). Extended elongation in domesticates compared to wild species suggests that elongation was a critical target for early selection of these plants, leading to longer fibers (Applequist et al., 2001; Kim, 2015). Many of these processes are regulated transcriptionally; domestication caused sweeping changes in the cotton transcriptome (Rapp et al., 2010; Gallagher et al., 2020). Domesticated species suppress lignin synthesis in the fiber, which is crucial to the development of the nearly-pure cellulose mature fiber (Tuttle et al., 2015). ROS regulation, signaling, and scavenging are all altered in the domesticates, indicating that these critical cell processes are relevant to the domesticated phenotype and potential targets for further improvement (Hovav et al., 2008; Chaudhary et al., 2009; Tang et al., 2014; Xu et al., 2019). Domestication also causes changes at the network level, with entire coexpression or regulatory networks changing under the selective pressure exerted during the domestication process, with networks becoming more tightly linked after domestication (in cotton seeds and fiber; Hu et al., 2016a; Gallagher et al., 2020; Hu et al., 2021). We are only at the beginning of understanding these phenomena and how they overlap and are distinct in each of the domesticated species. Continued study, therefore, promises new insights into the complex genotype to phenotype equation and the consequences of natural, and by extension, human-mediated selection. The diploid domesticates are understudied, and continuing to improve our knowledge of them also will provide opportunities for understanding the evolution and domestication of the cotton fiber, and the presumably critical role of polyploidy in facilitating the selection of vastly improved fiber. Taken together, this new knowledge will provide avenues of improvement that could lead to higher quality and yield of commercially grown cotton, either through deeper understanding of the domestication process, or through more traditional crop improvement techniques.

## 7 Conclusions

While *Gossypium* has been extensively studied for decades, our knowledge of how cotton fiber grows and develops remains in its infancy. A deeper understanding of the complex genetic, metabolic, and physiological underpinnings and networks that underlie fiber development would greatly benefit all those interested in cotton as a species. Increasing understanding of how plants can develop such a unique and specialized cell will also provide potential avenues for improving the most important textile crop in the world. The end of fiber development is of particular interest, due to the scarcity of research; understanding the mechanisms by which the fiber matures, ceases secondary wall synthesis, and ultimately dies could lead to finer control of these processes.

Understanding cotton fiber development is of agronomic interest as well, as it can lead to improvement of the world’s most prominent plant textile. A deeper understanding of the mechanisms behind fiber initiation and tip refinement could lead to enhancement of other textiles, or increase the yield or fineness of cotton fiber. A deeper knowledge of how cotton elongates fiber cells could provide insight into creating even longer fibers. The transition stage also has potential for improvement; adjustments to winding layer deposition could lead to stronger fibers. Fine tuning the shift from elongation and primary cell wall synthesis to fiber thickening and secondary cell wall deposition could result in higher quality fibers as well. Secondary wall synthesis itself is also a critical step in fiber development, and one that is heavily tied to overall fiber quality. Altering the thickness of the secondary cell wall at this stage could improve fiber quality.

Additionally, there is practical interest in understanding cotton fiber development in the context of a changing environment. As with most crops, cotton yield (i.e., boll production and retention) can be negatively influenced by climatic factors, including changes in heat accumulation and fluctuations in other abiotic stressors (Sawan, 2017a, Ullah et al., 2017; Sawan, 2017b; Kukal and Irmak, 2018; Saud and Wang, 2022; Sihi et al., 2022). Importantly, recent research suggests that environment plays a stronger role than genotype in determining agronomically fiber properties (e.g., yield, length, and uniformity) among elite lines of *G. hirsutum* (Raper et al., 2019). While some progress has been made in understanding the mechanisms of fiber production under stress and changing climatic conditions (Mishra et al, 2017; Ullah et al., 2017; Esmaeili et al., 2021), the mechanisms underlying the response of fiber development to environment is yet unknown. Therefore, understanding the nuances of cotton fiber development and how it is influenced by the environment is paramount in forward-thinking sustainability efforts.

Beyond the more practical applications of fiber improvement, studying the cotton fiber is itself valuable. It provides a ready-made system in which to study the development of a cell, situated in a polyploidy and domestication experiment that has been beautifully replicated over the history of the genus. The fibers of these four domesticated cotton species hold secrets of domestication, and how artificial selection impacts plants at the genomic, transcriptional, and developmental levels. They provide a unique opportunity to see how domestication can arrive at a similar phenotype across four independent domestication events. Cotton is ultimately an invaluable resource both as a textile crop and a system of study, and its fiber has the potential to unlock many mysteries across a range of disciplines.

## Author contributions

JJ and JW initiated the review and outlined the contents. JJ wrote the initial draft with insights from JW and CG. All authors participated in manuscript development and refinement. All authors contributed to the article and approved the submitted version.
